# Resistant Hypertension: Disease Burden and Emerging Treatment Options

**DOI:** 10.1007/s11906-023-01282-0

**Published:** 2024-02-16

**Authors:** John M. Flack, Michael G. Buhnerkempe, Kenneth Todd Moore

**Affiliations:** 1 Department of Medicine, Division of General Internal Medicine, Hypertension Section, Southern Illinois University, Southern Illinois University School of Medicine, 801 North Rutledge Street, Carbondale, IL 62702 USA; 2 Department of Medicine and the Center for Clinical Research, Southern Illinois University, Carbondale, IL USA; 3 Janssen Scientific Affairs, LLC, Titusville, NJ USA

**Keywords:** Blood pressure, Disease burden, Hypertension, Resistant hypertension

## Abstract

**Purpose of Review:**

To define resistant hypertension (RHT), review its pathophysiology and disease burden, identify barriers to effective hypertension management, and to highlight emerging treatment options.

**Recent Findings:**

RHT is defined as uncontrolled blood pressure (BP) ≥ 130/80 mm Hg despite concurrent prescription of ≥ 3 or ≥ 4 antihypertensive drugs in different classes or controlled BP despite prescription of  ≥ to 4 drugs, at maximally tolerated doses, including a diuretic. BP is regulated by a complex interplay between the renin–angiotensin–aldosterone system, the sympathetic nervous system, the endothelin system, natriuretic peptides, the arterial vasculature, and the immune system; disruption of any of these can increase BP. RHT is disproportionately manifest in African Americans, older patients, and those with diabetes and/or chronic kidney disease (CKD). Amongst drug-treated hypertensives, only one-quarter have been treated intensively enough (prescribed > 2 drugs) to be considered for this diagnosis. New treatment strategies aimed at novel therapeutic targets include inhibition of sodium-glucose cotransporter 2, aminopeptidase A, aldosterone synthesis, phosphodiesterase 5, xanthine oxidase, and dopamine beta-hydroxylase, as well as soluble guanylate cyclase stimulation, nonsteroidal mineralocorticoid receptor antagonism, and dual endothelin receptor antagonism.

**Summary:**

The burden of RHT remains high. Better use of currently approved therapies and integrating emerging therapies are welcome additions to the therapeutic armamentarium for addressing needs in high-risk aTRH patients.

## Introduction

As of 2018, 116 million US adults (47%) had hypertension, defined as blood pressure (BP) ≥ 130/80 mm Hg. Of these, 92 million Americans had uncontrolled hypertension because of inadequate medical treatment (34 million), lack of treatment despite a recommendation for medication (34 million), or unsuccessful management with lifestyle modifications alone (24 million) [1]. The global prevalence of hypertension is expected to rise from 972 million individuals (26.4% of the adult population) in 2000 to 1.56 billion (29.2%) by 2025—an increase of 60% [2]. Uncontrolled hypertension is the leading modifiable risk factor for cardiovascular disease (CVD), stroke, disability, and premature mortality worldwide [3], as well as the leading cause of mortality due to noncommunicable diseases [4]. Suboptimal management of hypertension remains a common public health problem despite the availability of effective and safe therapeutic regimens [3]. Patients with difficult-to-treat hypertension are often referred to as patients with “resistant” or “refractory” hypertension. Until recently, refractory hypertension historically has not been differentiated from resistant hypertension [5, 6]. This article reviews the definitions of resistant hypertension (RHT), the pathophysiology of RHT, patient characteristics and disease burden, current treatment guidelines for RHT, barriers to effective management, and emerging therapies for the treatment of RHT.

## What Is RHT?

### Definitions of RHT and Refractory Hypertension

Resistant hypertension is defined as either (1) uncontrolled BP remaining at ≥ 130 mm Hg systolic BP (SBP) and ≥ 80 mm Hg diastolic BP (DBP) despite the concurrent prescription of 3 or 4 antihypertensive drugs of different classes, or (2) controlled BP with the prescription of ≥ 4 antihypertensive drugs, with both definitions including a thiazide diuretic and all medications at maximally tolerated doses [3, 5, 7, 6, 7, 8••]. Uncontrolled cases of elevated BP in patients prescribed ≥ 3 adequately dosed antihypertensive drugs inclusive of a diuretic will include both RHT and refractory hypertension [9].

However, pseudo-resistance can often complicate the identification of RHT. Guidelines from the American College of Cardiology (ACC) and the American Heart Association (AHA) recommend 24-h ambulatory BP monitoring (ABPM) as the preferred BP monitoring method for excluding white-coat hypertension; however, ABPM is not widely used in contemporary clinical practice [10]. Secondary hypertension, in contrast to primary (essential) hypertension, is often associated with RHT and should be assessed before identification of true RHT [3]. Causes of secondary hypertension include kidney diseases, endocrine conditions (eg, Cushing syndrome, pheochromocytoma, hypothyroidism or hyperthyroidism, hyperparathyroidism, and primary aldosteronism), and other causes (eg, renovascular hypertension, coarctation of the aorta, obesity, and sleep apnea) [11]. Medication non-adherence also remains a common problem in hypertension treatment and can lead to overestimates of true treatment intensity when assessing RHT [3]. Patients fulfilling the aforementioned definitions of RHT in whom causes of pseudo-resistance cannot be ruled out – non-adherence to prescribed medications, secondary causes of hypertension, inaccurate BP measurement, white coat effect—are more appropriately referred to as having apparent treatment resistant hypertension (aTRH) [7]. We will use the term RHT and aTRH interchangeably in this review. The prevalence of apparent RHT is reported as 2% to 40% of hypertension patients in various studies [5, 12–14]; approximately 20% of US adults taking antihypertensive medication have apparent RHT according to the 2018 definition of the AHA [15]. Using data from the 1999–2020 National Health and Nutrition Examination Survey (NHANES), we found survey-weighted prevalence of resistant hypertension amongst drug-treated hypertensives to be 9.6% and 11.9% at the 140/90 mm Hg and 130/80 mm Hg BP thresholds, respectively [16]. The best estimates for the prevalence of refractory hypertension in adult hypertensives were reported by Buhnerkempe and coworkers [6] from an analysis that pooled data from 8 NHANES cycles (1999 – 2014) from 21,381 hypertensives; the prevalence of refractory hypertension was 0.6% in this national probability sample.

The importance of adequate treatment cannot be overstated. Many patients with uncontrolled BP are undertreated; they may be capable of control or, alternatively, may progress to RHT/refractory hypertension upon treatment intensification. Although 40% of drug-treated hypertensives are treated with a single antihypertensive drug [17•], the 2018 ACC/AHA hypertension guideline recommends starting with two evidence-based antihypertensive drugs when BP ≥ 140/90 mm Hg. Clinical inertia is defined as the failure of health care providers to initiate or intensify therapy when they encounter BP levels above goal and, in drug-treated hypertensives, compounds the initial prescription of inadequately intense drug therapy. Suboptimal patient adherence to prescribed treatments is also an important cause of uncontrolled BP; however, assessing patient adherence in most clinical settings remains challenging [4].

## Pathophysiology of RHT

Blood pressure is determined by complex interactions that occur among the renin–angiotensin–aldosterone system (RAAS), the sympathetic nervous system (SNS), the endothelin system, natriuretic peptides, the arterial vasculature, and the immune system [18]. Hypertension may result from dysfunction in any or all of these systems, with contributions from genetics, environmental factors (eg, high sodium intake, low potassium intake, sleep apnea, excessive alcohol intake, physical inactivity and stress), and aging.

### The Renin–Angiotensin–Aldosterone System

The RAAS is primarily responsible for BP control by regulating blood volume, sodium reabsorption, potassium excretion, water reabsorption, and vascular tone [19]. Juxtaglomerular cells in the kidney cleave inactive prorenin to renin in response to decreased BP or other stimuli. Renin is released into the bloodstream and converts angiotensinogen to angiotensin I, which is then converted to physiologically active angiotensin II via ACE. Angiotensin II binds to receptors in the kidney, adrenal cortex, arterioles, and brain and increases sodium reabsorption in the kidney, which increases blood osmolarity and moves fluid into the bloodstream and extracellular space, with an increase in arterial pressure. Vasoconstriction in systemic arterioles increases total peripheral resistance and BP. Angiotensin II also stimulates the release of aldosterone from the adrenal cortex; aldosterone binds to the mineralocorticoid receptor, regulates sodium/potassium balance in the kidney (increasing sodium absorption and potassium secretion), and alters gene transcription. Angiotensin also acts on the brain, stimulating thirst and water intake via the hypothalamus, effecting the release of antidiuretic hormone (vasopressin) by the pituitary to increase water reabsorption in the kidney, and decreases the baroreceptor response to an increase in BP [19].

Hypertension can arise from solitary or multiple perturbations in several interfacing physiologic systems. For example, critical renal artery stenosis causes a decline in blood flow within the kidney, which activates the RAAS to increase blood volume and arteriolar tone [19]. Inadequate systemic and renal arterial vasodilation in response to dietary sodium loading and plasma volume expansion (salt sensitivity), arterial stiffness, endothelial dysfunction, and SNS activation aggravate these processes and contribute to RHT. Secondary contributors comprise a variety of factors (eg, aging, aldosterone excess, inflammation, vascular calcification, poor BP control, drug resistance) as well as comorbidities (eg, obesity, diabetes, obstructive sleep apnea [OSA], and chronic kidney disease [CKD]) [20]. Arterial stiffness is associated with aging; kidney dysfunction contributes to antihypertensive drug resistance; diabetes contributes to CKD; and CKD contributes to endothelial dysfunction, vascular calcification, and arterial stiffness [20]. OSA is linked to high aldosterone levels and RHT; these linkages may be partially mediated by increased sympathetic tone [7, 21].

### Natriuretic Peptides

Atrial natriuretic peptide and brain natriuretic peptide play an important role in maintaining balanced salt levels and normal BP. Sodium loading results in release of atrial natriuretic peptide and brain natriuretic peptide, which promotes BP lowering through systemic vasodilation and decreased plasma volume due to fluid diversion from the intravascular to the interstitial compartment [18]. Natriuretic peptides also increase glomerular filtration rate (GFR) and inhibit renal sodium reabsorption.

### Sodium Homeostasis and the Endothelium

Normally, high dietary sodium intake sets in motion compensatory hemodynamic changes to maintain constant BP, including release of the vasodilator nitric oxide from the endothelium with subsequent decreases in both renal and peripheral vascular resistance. However, when endothelial dysfunction (eg, a suboptimal vasodilatory response or decrease in peripheral resistance in response to salt loading) is present, especially with concurrent low dietary potassium intake, patients can develop salt sensitivity and, subsequently, hypertension [18, 22]. Likewise, loss of nitric oxide production through inhibition of endothelial nitric oxide synthase impairs smooth muscle relaxation and leads to arterial stiffness and increased systemic vascular resistance.

Another pathophysiologic mechanism for hypertension involves the endothelin system, specifically endothelin-1 (ET-1), produced in the vascular endothelium [23, 24]. Under normal conditions, production and clearance of ET-1 are balanced, but in diseases such as pulmonary arterial hypertension (PAH), circulating levels of ET-1 rise, with detrimental effects [25]. ET-1 regulates vascular tone and BP and contributes to hypertension via vasoconstriction, vascular hypertrophy and remodeling, neurohormonal and sympathetic activation, increased aldosterone secretion, endothelial dysfunction, and end-organ damage [26]. In addition to vasoconstriction and growth of smooth muscle cells, ET-1 activation enhances the production of growth factors and inflammatory mediators; upregulates adhesion molecules, chemokines, and cytokines; and causes atherosclerosis, fibrosis, and vascular damage [23]. ET-1 therefore contributes to CVD, PAH, CKD, ischemic heart disease, and stroke [23, 24, 27]. ET-1 expression is upregulated in severe hypertension as well as in salt-sensitive hypertension, obesity, diabetes, and CKD [26, 28].

The actions of ET-1 are mediated by 2 G-protein–coupled receptors, ET_A_ and ET_B_. ET_A_ receptors are expressed on pulmonary smooth muscle cells and mediate vasoconstriction and cellular proliferation. ET_B_ receptors are expressed mainly on the endothelial surface of vessels and mediate vasodilation by producing nitric oxide and prostacyclin; however, in hypertension and other cardiovascular diseases, they are also upregulated in vascular smooth muscle cells, where they have the same pathophysiologic actions as ET_A_ receptors [24].

### Sympathetic Nervous System

Heightened SNS activity, possibly coupled with decreased parasympathetic nervous system activity, causes BP elevations via a multiplicity of physiological actions including vasoconstriction, arterial stiffness, endothelial dysfunction as well as by contributing to salt sensitivity [18]. The SNS also regulates BP through the action of baroreceptors that sense pressure changes in selected sites in the circulatory system. Normally, baroreceptors respond to arterial stretching (resulting from elevated BP) by signaling the brain to reduce the sympathetic outflow of nerve impulses, thereby lowering BP [18]. SNS hyperactivity and/or SNS activity excess relative to parasympathetic nervous system activity, has been associated with both the initiation and maintenance of hypertension. Data show that patients with hypertension have greater SNS activity than normotensive individuals, especially amongst those who are obese, and the level of sympathetic activity is positively correlated with the severity of hypertension [18].

### Immune System

Inflammation contributes to hypertension through an increase in vascular permeability and the release of reactive oxygen species, cytokines, and metalloproteinases [18]. Cytokines contribute to intimal thickening and vascular fibrosis/stiffness, while matrix metalloproteinases promote extracellular matrix degradation, immune cell infiltration, apoptosis, and excess collagen synthesis. The innate and adaptive immune systems generate reactive oxygen species and can cause inflammation in the blood vessels, kidneys, and brain. The innate immune system can contribute to hypertension via dysregulation of angiotensin II, aldosterone, and nitric oxide function. Within the adaptive immune system, imbalances between proinflammatory T cells and regulatory T cells that suppress inflammation have been implicated in the development of hypertension and hypertension-induced kidney disease.

## Patient Characteristics and Disease Burden

Patients with RHT have distinct phenotypic tendencies. When compared with patients who have nonresistant hypertension, patients with RHT are significantly more likely to be older, obese (body mass index ≥ 30 kg/m^2^), of Black race (versus non-Black), and male, and also have a higher prevalence of comorbid conditions, including diabetes, ischemic heart disease, prior stroke, and CKD/albuminuria [6, 29]. Common comorbidities and lifestyle choices elevate BP, including obesity, diabetes, salt sensitivity, poor dietary habits, excess sodium and inadequate potassium intake, smoking, alcohol use, and physical inactivity [3, 30]. Many of these characteristics are interrelated; for example, salt sensitivity, which is found in more than half of all US adults, is common among Black individuals, older patients, and those with comorbidities such as diabetes, metabolic syndrome, and CKD [30].

Inadequate RHT treatment leads to elevated risk of major adverse cardiovascular events and end-organ damage with associated morbidity and mortality [3]. Patients are also at risk of nonalcoholic fatty liver disease, which shares biologic processes and causative genes with hypertension [31]. The risk of cognitive impairment and dementia is elevated because of hypertension-associated white-matter lesions [32]. Disease burden of hypertension increases from undertreated to uncontrolled to resistant to refractory hypertension, with rising prevalence of medication adverse effects, target organ damage, morbidity, and mortality [3, 7, 8••, 9]. Retrospective US data showed a 47% higher risk of death due to CVD among patients with apparent RHT compared with nonresistant hypertension after control for confounding factors, and risk of CVD death was significantly higher regardless of hypertension control [33]. Risk of myocardial infarction, heart failure, stroke, and renal failure rise along with the severity and duration of hypertension. A bidirectional relationship exists whereby structural and functional changes in the cardiovascular system due to RHT (eg, aortic stiffness, atherosclerosis, renal dysfunction) as well as comorbidities (eg, obesity, diabetes, hyperaldosteronism) may make hypertension harder to control [9].

Subsets of patients have higher disease burden than others because of socioeconomic or treatment-related factors. Socioeconomic factors that affect treatment use and success (as well as adherence) include minority race/ethnicity, low income, lack of insurance, low literacy, poor disease understanding, and inadequate transportation [4]. Poor medication access and affordability can negatively influence clinical outcomes [4]. Uninsured adults with hypertension were found to have lower disease awareness, lower treatment rates, and lower rates of hypertension control than insured adults; these 3 factors were estimated to cause a 22% absolute gap in BP control [34]. Prescription of thiazide-like diuretics (jndapamide, chlorthalidone) and MRAs (spironolactone, eplerenone) is underutilized in Black and White individuals with aTRH [6, 32].

Spending on hypertension was estimated at approximately $131 billion annually in the United States as of 2014 [35]. Compared with nonresistant hypertension, RHT is associated with an added economic burden on both the US health care system and individual patients. Total annual US health care (medical and prescription) expenditures and health care utilization rates are significantly greater for apparent RHT than for nonresistant hypertension [36]. Apparent RHT is estimated to increase US health care expenditures by $11 billion to $18 billion per year over those associated with non-resistant hypertension [36].

## Current Approved Therapies for RHT

**Fig. 1:**
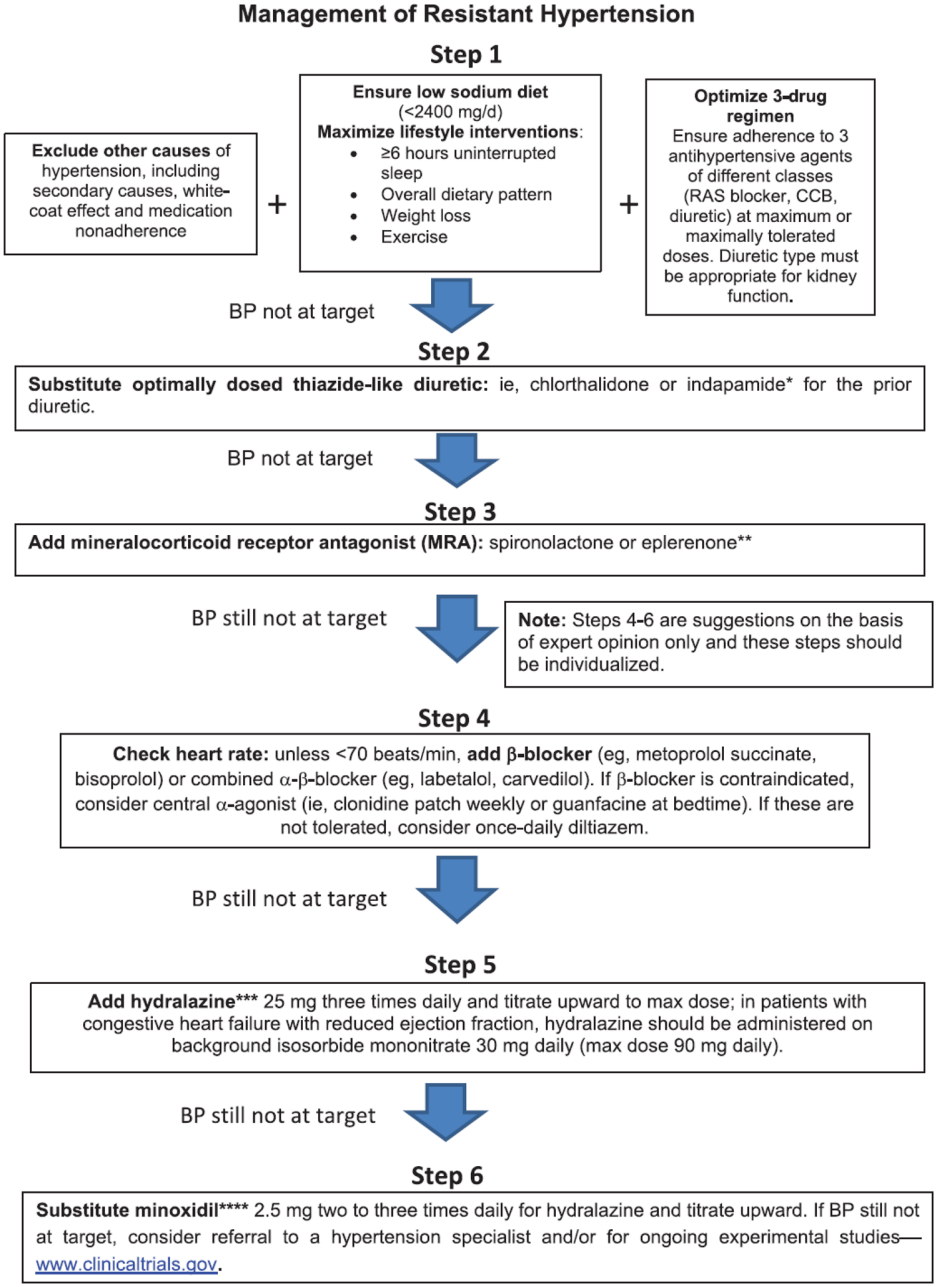
Management of Resistant Hypertension Reprinted with permission. Hypertension. 2018;72(5):e53-e90. ©2018 American Heart Association, Inc. RAS, renin-angiotensin system; CCB, calcium channel blocker; BP, blood pressure.

Treatment of RHT proceeds in a stepwise fashion (Fig. 1). After excluding causes of pseudo-resistance to the degree possible, Step 1 consists of both lifestyle interventions and medical treatment. Lifestyle interventions include weight loss, exercise, a low-sodium diet (< 2000 mg/day), augmented dietary potassium intake, and/or the Dietary Approaches to Stop Hypertension (DASH) diet; these interventions are implemented in addition to adherence with a 3-drug regimen with 3 antihypertensive agents of different classes, including a RAS blocker, a CCB, and a diuretic at maximum tolerated doses [3, 30, 37]. If RHT is not controlled, Step 2 consists of an optimally dosed and more potent thiazide-like diuretic (chlorthalidone or indapamide) in place of hydrochlorothiazide if the latter was initially used [3].

If hypertension has been treated intensively enough (≥ = 3 drugs inclusive of a diuretic), the patient meets criteria for RHT or refractory hypertension. Then, a logical treatment escalation strategy proceeds to Step 3, in which an MRA (spironolactone or eplerenone) is added if kidney function is adequate (ie, estimated GFR > 45 mL/min/1.73 m^2^) [3, 37]. Amiloride may be considered as an alternative to spironolactone [7, 37]. After Step 3, all subsequent treatment steps are individualized and optimized (Fig. 1). However, it should be noted that this approach ignores likely comorbid conditions such as heart failure, CKD and prior myocardial infarction. When selected comorbidities (eg, post-MI) are present, the highest priority drug(s) (eg, beta blocker) for inclusion into the drug regimen are those that are indicated for the specific comorbidities [30].

## Treatment Barriers

Although controlled clinical trials demonstrate the possibility of excellent control of hypertension, rates of BP control in community settings are often disappointing [38]. Several potential reasons explain why guidelines for RHT treatment often are not followed. Importantly, treatment guidelines are long and complex, and many physicians do not read them in detail [39]. Other treatment barriers involve those related to patients (eg, poor adherence and persistence with prescribed treatments, lack of access to a usual site of care, or poor health literacy) as well as obstacles related to the clinician and health care system (eg, therapeutic inertia, poor communication with patients, and fragmentation of care) [3, 38]. Therapeutic inertia (or clinical inertia) increases the likelihood of not attaining goal BP levels [40]. It is estimated that therapeutic inertia occurs in 75% of medical consultations for hypertension [41]. Potential reasons for the high rates of RHT reported in some studies may relate to prescription of ineffective drug combinations, the lack of up-titration of antihypertensive drugs in patients already prescribed several drugs when BP is elevated, and non-adherence to prescribed drug therapy [3, 7, 37]. Overall, BP control in the United States deteriorated from 2015 to 2018 (compared with 2009 to 2014) because of declines in patient awareness, treatment receipt, and treatment effectiveness. The use of monotherapy increased during this interval, and nearly three-quarters (74%) of patients were receiving only 1 to 2 antihypertensive medications from 2015 to 2018 [42]. Declines in BP control were also noted from 2017 to 2020, when only 48% of all US adults with hypertension were considered controlled [43].

Medication nonadherence is a common problem among patients with RHT. In a study of 108 patients with RHT (uncontrolled despite use of ≥ 4 antihypertensive medications), 53% were found to be nonadherent; of these, 30% were completely nonadherent and 70% were incompletely nonadherent [44]. Reasons include adverse drug effects, pill burden, drug costs, long duration of treatment, and lack of communication from clinicians about the importance of medication adherence and persistence [3, 4, 38]. Because adherence declines as the number of medications rises and the treatment regimen becomes more complex, poor adherence is a particular problem complicating the treatment of patients with RHT [7]. Patients often poorly understand the consequences of hypertension and possible adverse effects of medications and may also have low health literacy in general [3, 4]. Some patients may perceive that their prescription antihypertensive therapies are ineffective for BP control [4]. Patients often have medical comorbidities, psychological difficulties (depression, dementia, and substance abuse), and social disadvantages such as poverty that can hinder attainment of BP control [4]. Regardless of the reasons, suboptimal adherence to prescribed antihypertensive therapy is a major contributor to inadequate BP control and thus increases the risk of major adverse cardiovascular events, including myocardial infarction, stroke, chronic heart failure, CKD, and death [4]. Suboptimal adherence to prescribed medications also increases total health care costs [4].

## Current Research Into New Treatments and Modalities

### Limitations of Current Treatments

In addition to the management challenges of treatment inertia and patient nonadherence, all drug classes for hypertension have certain drawbacks. Adverse effects occur with ACE inhibitors (eg, cough, angioedema, and hyperkalemia), ARBs (eg, hyperkalemia), and CCBs (eg, peripheral edema, flushing, tachycardia, dizziness, and cardiac depression) [45]. Diuretics are also associated with adverse effects (eg, electrolyte imbalances, hyperglycemia, hyperlipidemia, and hyperuricemia); however, these are often mitigated by addition of a RAS blocker [45]. Beta-blockers can cause drowsiness, sleep disturbance, depression, visual hallucinations, erectile dysfunction, and peripheral vascular side effects [45]. Spironolactone has good efficacy and is recommended as the first choice of fourth-line agent; however, the risk of hyperkalemia precludes its use in patients with CKD and an estimated GFR < 45 mL/min/1.73 m^2^ or in those with serum potassium > 4.5 mEq/L [3, 46]. Treatment of OSA-associated hypertension with continuous positive airway pressure (CPAP) is shown to reduce SBP by only about 2 to 5 mm Hg, and any improvement depends on patient adherence with its use [3].

### Device-based Therapies for RHT

If medical therapy is ineffective, device-based interventions may ultimately be an option for adjunctive treatment; these treatments include renal denervation (RDN) and baroreceptor stimulation [37]. The rationale for RDN is based on the role of the SNS in hypertension via renal vascular resistance, renin release, and sodium reabsorption [37]. Minimally invasive denervation can be achieved via catheter-based application of radiofrequency, ultrasound, or injection of neurotoxic agents [37]. Two RDN devices were granted breakthrough therapy designation by the US Food and Drug Administration (FDA) in December 2020 [47]. However, the magnitude of the RDN treatment effect, average BP reductions of < 10 mm Hg, suggest that this permanent approach to lowering BP will be most useful in combination with, not in lieu of, antihypertensive drugs; no devices have been approved by the FDA [3, 12, 37, 48].

Late-breaking results from a trial of radiofrequency RDN were reported at the 2022 AHA Scientific Sessions held in November 2022 [49]. The SPYRAL HTN-ON MED trial was an international, sham-controlled, randomized trial of patients with office SBP ≥ 150 to < 180 mm Hg who were stable on up to 3 antihypertensive (thiazide diuretic, CCB, ACE inhibitors, and β-blockers) medications for 6 weeks at baseline. The primary efficacy endpoint of the study evaluated changes in 24-h SBP by ambulatory BP monitoring after 6 months of study treatment, comparing RDN and sham control using a Bayesian analysis with a success threshold of 97.5%; RDN did not meet the primary efficacy endpoint (treatment difference, –0.03 mm Hg; 95% confidence interval [CI], –2.82 to 2.77). Despite the lack of significant change in 24-h ambulatory SBP and DBP, office measures of SBP and DBP were significantly decreased with RDN versus sham control (SBP, –9.9 vs –5.1 mm Hg; *P* = 0.001; DBP, − 5.2 vs –3.3 mm Hg; *P* = 0.04). The sham group also had significantly higher antihypertensive medication use during follow-up. Notably, 80% of patients had trial follow-up during the COVID-19 pandemic, and significant differences were detected when comparing 24-h ambulatory SBP, but not office SBP, in measurements collected before and during the pandemic. These differences may plausibly have affected the trial conclusions. Safety outcomes indicated a low incidence of procedure-related and clinical adverse events with RDN.

Ultrasound RDN (uRDN) has been evaluated in the multinational, randomized, single-blind, sham-controlled RADIANCE-HTN TRIO trial in patients with office measured ≥ 140 mmHg SBP and ≥ 90 mmHg DBP despite stable doses of 3 or more antihypertensives, including a diuretic [50]. At enrollment, patients were switched to fixed dose amlodipine, valsartan or olmesartan, and hydrochlorothiazide. For the primary endpoint of change from baseline to 2 months in daytime ambulatory SBP, uRDN demonstrated a significantly greater reduction than sham control (median, –8.0 mmHg [IQR, –16.4 to 0.0] vs –3.0 mmHg [IQR, –10.3 to 1.8]; median between-group difference, –4.5 mm Hg [95% CI, –8.5 to –0.3]; baseline adjusted *P* = 0.022). Reduction in 24-h ambulatory SBP was also significantly greater with uRDN versus sham control. Between baseline and 2 months, a similar percentage of patients in the uRDN and sham control groups had no change in their baseline antihypertensive treatment (93% vs 85%; *P* = 0.15). In the prespecified 6-month analysis of RADIANCE-HTN TRIO, the change from baseline in mean daytime ambulatory SBP was not significantly different between uRDN and sham control (mean difference, –2.5 mmHg [95% CI –6.7 to 1.7]; *P* = 0.25). The fixed-dose combination antihypertensive therapy remained unchanged at 6 months in both groups (76.9% and 82.8% in the uRDN and sham control groups, respectively); however, fewer antihypertensive medications were added in the uRDN group versus the sham control group.

Carotid baroreceptor activation therapy involves the use of an implanted pulse generator connected to leads placed next to the carotid sinus [3]. The device stimulates baroreceptors, which signal the brain to reduce sympathetic overactivity, which in turn reduces heart rate and cardiac workload, dilates the arteries, improves renal blood flow and sodium excretion, and decreases BP [3]. Baroreceptor activation therapy has been approved for the treatment of advanced heart failure [3, 51], but not as yet for use in hypertension. Nevertheless, this is a more invasive procedure than RDN that leaves the patient with internally implanted hardware.

### New Pharmacologic Options

New pharmacologic options for RHT are being investigated in clinical trials, targeting both well-known disease pathways and recently elucidated pathophysiologic mechanisms (Table 1) [46]. Emerging drug classes include sodium–glucose cotransporter 2 inhibitors (eg, empagliflozin, canagliflozin, and dapagliflozin), which were approved for glycemic control in type 2 diabetes and have also been shown to reduce BP and CVD-renal events [45, 51, 52]. Neprilysin, a membrane-bound zinc endopeptidase that degrades natriuretic peptides, has been targeted in dual angiotensin receptor–neprilysin inhibitor drugs (eg, sacubitril/valsartan). This drug combination enhances natriuresis and vasodilatation and reduces BP, arterial stiffness, cardiac hypertrophy, and fibrosis [52]. Neprilysin inhibitors are also being evaluated as add-on therapy to multidrug regimens as well as in combination with other agents [46].

**Table 1:** Summary of recent clinical trials of investigational agents in hypertension

Drug (brand name)/manufacturer	Mode of action	Development status	Trial name/ identifier	Study population enrollment criteria	N	Primary/secondary BP endpoints	Primary/secondary BP endpoint results
Dapagliflozin (Farxiga^®^)/AstraZeneca [61]	SGLT2 inhibitor	Phase 3	NCT01195662	Patients with T2DM and seated SBP 140–165 mmHg and DBP 85–105 mmHg on a renin-angiotensin blocker and 1 other antihypertensive medication	449	Change from baseline to Week 12 in mean seated SBP	Dapagliflozin 10 mg –11.90 mmHg (95% CI, –13.97, –9.82) vs placebo –7.62 mmHg (95% CI, –9.72, –5.51); placebo-adjusted treatment difference –4.28 mmHg (95% CI, –6.54, –2.02), *P* = 0.0002
Empagliflozin (Jardiance^®^)/Boehringer Ingelheim [62]	SGLT2 inhibitor	Phase 3	NCT01370005 (EMPA-REG BP)	Patients with T2DM and mean seated office SBP 130–159 mmHg and DBP 80–99 mmHg on ≤ 2 antihypertensive medications	823	Change from baseline to Week 12 in mean 24-h ambulatory SBP	Empagliflozin 10 mg –2.95 mmHg vs placebo + 0.48 mmHg; mean treatment difference, –3.44 mmHg (95% CI –4.78, –2.09), *P* < 0.001; empagliflozin 25 mg –3.68 mmHg vs placebo + 0.48 mmHg; mean treatment difference, –4.16 mmHg (95% CI, –5.50, –2.83), *P* < 0.001
Empagliflozin (Jardiance^®^)/Boehringer Ingelheim [63]	SGLT2 inhibitor	Phase 3	NCT01131676 (EMPA-REG OUTCOME)	Post hoc analysis of patients with T2DM, established CVD and seated office SBP ≥ 140 mmHg and/or DBP ≥ 90 mmHg on ≥ 3 classes of antihypertensives, or SBP < 140 mmHg and DBP < 90 mmHg on ≥ 4 classes of antihypertensives	1579	Change from baseline to Week 12 in mean office seated SBP and DBP	Empagliflozin vs placebo, mean treatment difference: SBP –4.5 mmHg (95% CI –5.9, –3.1); DBP –1.7 mmHg (95% CI, –2.5, –0.9)
Canagliflozin (Invokana®)/Janssen [64]	SGLT2 inhibitor	Phase 4	NCT01939496	Patients with T2DM and seated office SBP 130–160 mmHg/DBP ≥ 70 mmHg on 1–3 antihypertensives	169	Change from baseline to Week 6 in mean 24-h ambulatory BP	Canagliflozin 300 mg: SBP –6.2 mmHg (SE, 1.4) vs placebo –1.2 mmHg (SE, 1.4), *P* = 0.006; DBP –3.2 (SE, 0.8) vs placebo –0.3 mmHg (SE, 0.8), *P* = 0.005; canagliflozin 100 mg: SBP –4.5 mmHg (SE 1.4) vs placebo –1.2 mmHg (SE, 1.4), *P* = 0.062; DBP –2.2 mmHg (SE, 0.8) vs placebo –0.3 mmHg (SE 0.8), *P* = 0.062
Bexagliflozin (Brenzavvy^®^)/Theracos [65]	SGLT2 inhibitor	Phase 2/3	NCT03514641	Patients with essential hypertension: office seated SBP 140–179 mmHg; unmedicated or on ≤ 4 antihypertensives	673	Change from baseline to Week 12 in mean 24-h SBP (Phase 2); change from 12 to 24 weeks in mean 24-h SBP (Phase 3)	Trial completed but results not yet reported
Firibastat/Quantum Genomics SA [53]	Aminopeptidase A inhibitor	Phase 3	NCT04277884 (FRESH)	Patients with RHT on ≥ 3 antihypertensives (including a diuretic)	515	Change from baseline to Week 12 in unattended digital clinic SBP	Firibastat 500 mg BID: –7.82 mmHg vs placebo: –7.85 mmHg; *P* = 0.98
Baxdrostat/CinCor Pharma [54]	Aldosterone synthesis inhibitor	Phase 2	NCT04519658 (BrigHTN)	Patients with RHT on ≥ 3 antihypertensives and mean seated BP ≥ 130/80 mmHg	275	Change from baseline to Week 12 in mean seated SBP	Baxdrostat 2 mg: –20.3 mmHg (SE, 2.1) vs placebo: –9.4 mmHg, *P* < 0.001; baxdrostat 1 mg: –17.5 mmHg (SE 2.0), vs placebo: –9.4 mmHg, *P* = 0.003; baxdrostat 0.5 mg: –12.1 (SE, 1.9) vs placebo: –9.4 mmHg, *P* = NS
Esaxerenone (Minnebro^®^)/Daiichi Sankyo [66]	Nonsteroidal MRA	Phase 3	NCT02890173 (ESAX-HTN)	Patients with essential hypertension: seated SBP 140–179 mmHg and DBP 90–109 mmHg	994	Change from baseline to Week 10–12 in mean seated BP	Esaxerenone 5 mg SBP: –16.9 mmHg vs eplenerone –12.1 mmHg, mean treatment difference: –4.8 mmHg (95% CI, –6.4, –3.1), *P* < 0.0001; esaxerenone 5 mg DBP: –8.4 mmHg vs eplenerone –6.1 mmHg, mean treatment difference: –2.3 (95% CI, –3.3, –1.3), *P* < 0.0001; esaxerenone 2.5 mg SBP: –13.7 mmHg vs eplenerone –12.1 mmHg, mean treatment difference: –1.6 mmHg (95% CI, –3.3, 0.1), *P* = 0.0709; esaxerenone 2.5 mg DBP: –6.8 mmHg vs eplenerone –6.1 mmHg, mean treatment difference: –0.7 (95% CI, –1.8, 0.3), *P* = 0.1601
Praciliguat/Cyclerion Therapeutics [67]	sGC stimulator	Phase 2A	NCT03091920	Patients with T2DM and SBP 120–160 mmHg/DBP 70–100 mmHg on an ACE inhibitor or ARB	26	Change from baseline to Week 2 in mean 24-h ambulatory BP^a^	Mean treatment difference praliciguat vs placebo: SBP: –2 mmHg (95% CI, –10, 5); DBP: –4 mmHg (95% CI –9, 1)
Sildenafil (Revatio^®^)/Pfizer [68]	PDE5 inhibitor	Phase 2	NCT00317421	Patients with untreated hypertension: SBP ≥ 160 mmHg or DBP ≥ 100 mmHg, mean ambulatory daytime SBP ≥ 145 mmHg or DBP ≥ 95 mmHg, or SBP 135–144 mmHg or DBP 85–94 mmHg and 10-year risk of CVD > 20%	22	Change from baseline to Day 16 in 24-h ambulatory SBP and DBP	SBP: sildenafil –7 mmHg (SE, 1.9) vs placebo 3 mmHg (SE, 2.1), *P* < 0.01; DBP: sildenafil –5 mmHg (SE, 1.1) vs placebo 1 mmHg (SE, 1.3), *P* < 0.01
Allopurinol/ University of Florida [69]	Xanthine oxidase inhibitor	Phase 3	NCT00241839	African-American patients with stage 1 hypertension on chlorthalidone	110	Change from baseline to Week 4 in seated office SBP	Allopurinol: –3.4 mmHg (SD, 12.25) vs placebo: 0.8 mmHg (SD 10.63); mean treatment difference –4.3 mmHg; *P* = 0.059
Etamicastat [70]	Dopamine beta-hydroxylase inhibitor	Phase 2A	EudraCT 2008–002789-09	Male patients with essential hypertension: supine SBP 140–179 mmHg and/or DBP 90–109 mmHg	23	Change from baseline to 10 days in mean 24-h ambulatory BP^a^	Mean treatment difference vs, placebo, etamicastat 50 mg SBP: –7.11 mmHg (95% CI, –18.64 to 4.42), DBP: –4.65 mmHg (95% CI, –12.08, 2.78); Etamicastat 100 mg SBP: –9.06 mmHg (95% CI, –20.37, 2.24), DBP: –5.57 mmHg (95% CI, –12.67, 1.54); etamicastat 200 mg SBP: –9.53 mmHg (95% CI –20.52, 1.47), DBP: –5.48 mmHg (95% CI, –12.13, 1.17)
Nesiritide/Oslo University Hospital [71]	Recombinant B-type natriuretic peptide	Phase 1/2	NCT02608996 (TENSE1)	Patients on ≥ 1 antihypertensive with office SBP ≥ 120 mmHg, and mean daytime ambulatory SBP > 115 mmHg	15 (planned)	Change in office BP and ambulatory BP over 48 h	Trial ongoing – estimated completion 2030
Zilebesiran/Alnylam Pharmaceuticals [72]	siRNA angiotensinogen reducer	Phase 1	NCT03934307	Patients with treated or untreated hypertension: mean seated SBP 130–165 mmHg	107	Change from baseline to Week 24 in mean 24-h ambulatory SBP and DBP^b^	Zilebesiran 200 mg: SBP –12.5 mmHg, DBP –5.7 mmHg; zilebesiran 400 mg: SBP –9.3 mmHg, DBP –5.4 mmHg; zilebesiran 800 mg: SBP –22.5 mmHg, DBP –10.8 mmHg
Aprocitentan/Idorsia Pharmaceuticals [60•]	Dual endothelin antagonist	Phase 3	NCT03541174 (PRECISION)	Patients with seated unattended office SBP ≥ 140 mmHg on ≥ 3 AHAs from different classes	730	Change from baseline to Week 4 in mean trough seated office SBP	Aprocitentan 12.5 mg –15.3 mmHg (SE, 0.9), mean difference vs placebo –3.8 mmHg (97.5% CI, –6.8, –0.8), *P* = 0.0042; aprocitentan 25 mg –15.2 mmHg (SE, 0.9), mean difference vs placebo –3.7 mmHg (97.5% CI –6.7, –0.8), *P* = 0.0046

^a^Secondary endpoint.

^b^Exploratory endpoint

AGE advanced glycation end products, CI confidence interval, CVD cardiovascular disease, DBP diastolic blood pressure, MRA mineralocorticoid receptor antagonist, NS not significant, PDE5 phosphodiesterase 5, SBP systolic blood pressure, SD standard deviation, SE standard error, sGC soluble guanylate cyclase, SGLT2 sodium-glucose cotransporter 2, siRNA small interfering ribonucleic acid, T2DM type 2 diabetes mellitus

Firibastat is an investigational oral agent that inhibits the action of aminopeptidase A in the brain, thereby blocking conversion of angiotensin II to angiotensin III [45, 51]. Late-breaking results of the phase 3 Firibastat in Treatment-resistant Hypertension (FRESH) trial were reported at the 2022 AHA meeting [53]. Participants had uncontrolled primary hypertension despite > 80% adherence to treatment with 2 classes of drugs (for difficult-to-treat hypertension) or ≥ 3 classes including a diuretic (for RHT) and were treated with firibastat 500 mg twice daily or placebo. The primary efficacy endpoint (ie, the change from baseline in unattended automated office SBP after 12 weeks of treatment) was not met (firibastat –7.82 mm Hg vs placebo –7.85 mm Hg; *P* = 0.98). Secondary endpoints, including ambulatory BP monitoring, also showed no significant differences between firibastat and placebo.

Aldosterone synthase inhibitors were developed to counteract the reactive upregulation of the RAAS and SNS. The BrigHtn study, a phase 2, randomized, double-blind, placebo-controlled, multicenter dose-ranging study, evaluated BP lowering effects of the aldosterone synthase inhibitor baxdrostat [54]. Participants had a seated BP of ≥ 130/80 mm Hg and were ≥ 70% adherent to a stable regimen of ≥ 3 antihypertensive agents (including a diuretic) for ≥ 4 weeks before randomization. The primary endpoint was change from baseline in mean seated SBP at 12 weeks with baxdrostat (0.5 mg, 1 mg, or 2 mg) versus placebo. Baxdrostat showed a statistically significant reduction in placebo-corrected SBP at the 2-mg dose (–11.0 mm Hg; *P* < 0.001) and the 1-mg dose (–8.1 mm Hg; *P* = 0.003). Baxdrostat 2 mg also showed a reduction in the secondary endpoint of placebo-corrected DBP (–5.2 mm Hg). Treatment was well tolerated, with no serious adverse events considered related to the drug. Laboratory measures up to day 85 confirmed a dose-dependent reduction in 24-h urinary and serum aldosterone, an increase in plasma renin activity, and no reduction in serum cortisol, all of which support the selective mechanism of action of baxdrostat.

Other novel agents under investigation for the treatment of RHT include nonsteroidal MRA agents (eg, esaxerenone), soluble guanylate cyclase stimulators (eg, praliciguat), phosphodiesterase-5 inhibitors (eg, sildenafil), xanthine oxidase inhibitors (eg, allopurinol), dopamine beta-hydroxylase inhibitors (eg, etamicastat), recombinant B-type natriuretic peptide (nesiritide), and advanced glycation end-product breakers (eg, alagebrium) [45, 46, 52]. Vaccines targeted against angiotensin I or II may prove viable in the future as a means to decrease SBP without the need for patient adherence to complex drug regimens [46, 52]. In a phase 1 trial, the pharmacokinetic and pharmacodynamic profiles of single ascending doses of the small interfering RNA therapeutic zilebesiran, which inhibits hepatic angiotensinogen synthesis, have been evaluated in a small number of patients with hypertension [55]. In exploratory endpoint analyses, decreases from baseline in SBP and DBP were observed after 8 weeks of treatment (SBP, –22.5 [SE, 5.1] mmHg; DBP, –10.8 [SE, 2.7] mmHg). A phase 2 trial of zilebesiran in patients with hypertension is ongoing.

### Endothelin Receptor Antagonists

As discussed earlier, the endothelin system of vasoconstrictor peptides plays a role in the pathophysiology of hypertension, primarily via ET-1, which was discovered to be the most potent known endogenous vasoconstrictor [23, 24, 52]. ET-1 is produced in the vascular endothelium and exerts its actions via 2 receptors, ET_A_ and ET_B_ [23, 24]. ET_A_ receptors primarily mediate smooth muscle cell contraction, whereas ET_B_ receptors have a similar function in smooth muscle but also can mediate vascular dilatation in endothelial cells through nitric oxide release [25]. Endothelin receptor antagonists (ERAs) offer either selective ET_A_ blockade or dual blockade against both ET_A_ and ET_B_ receptors [24, 26]. The rationale for selective ET_A_ blockade is to maintain the potential beneficial effects of ET_B_ receptors [24], while the rationale for dual blockade is to suppress the function of ET_A_ receptors plus the pathophysiologic actions of ET_B_ receptors [24, 25]. Research has confirmed that dual receptor blockade improves vascular remodeling more effectively than selective blockade while causing fewer adverse effects [56].

Initial efforts to develop this drug class included ERA agents ambrisentan (an ET_A_-selective agent) and bosentan and macitentan (both dual ET_A_ and ET_B_ antagonists) [24]. The first ERA studied in humans was bosentan, which significantly reduced DBP versus placebo in essential hypertension, but safety concerns arose regarding hepatotoxicity [24, 26]. Ambrisentan significantly improved exercise capacity in patients with PAH compared with placebo, with adverse effects of peripheral edema, headache, and nasal congestion, but with a lower risk of hepatic injury than with bosentan [24, 57]. Macitentan was the first dual-receptor ERA to demonstrate significant decreases in morbidity and mortality among patients with PAH in a phase 3 trial, with an improved hepatic safety profile compared with bosentan and less fluid retention than with ambrisentan [24, 25]. Darusentan, a selective ET_A_ receptor antagonist, failed to meet the primary efficacy endpoint of improvement in SBP after 14 weeks compared to placebo or guanfacine in patients with RHT and had an unfavorable safety profile [58]; further studies with darusentan were not conducted [26, 45]. Research into the ERA drug class was continued because of conflicting results regarding BP lowering, adverse effects of fluid retention, and deficiencies in trial design and patient selection in previous studies [26, 59, 60•].

Aprocitentan is an oral dual ERA that acts on both the ET_A_ and ET_B_ receptors and is the active metabolite of macitentan [56]. Phase 2 study results showed superior BP lowering with aprocitentan versus placebo or lisinopril in essential hypertension, leading to a pivotal phase 3 trial in patients with RHT [27]. The PRECISION trial was a blinded, randomized, phase 3 study designed to evaluate the effectiveness of aprocitentan when added to standard care for reducing BP compared with placebo over 48 weeks in patients with RHT, excluding patients with apparent or pseudo-resistant hypertension [59, 60•]. RHT was verified by a history of uncontrolled BP despite compliance with ≥ 3 antihypertensive medications of different drug classes for ≥ 1 year, SBP ≥ 140 mm Hg at screening, and no known secondary causes of hypertension [59, 60•]. The study design comprised a 4-week, double-blind, randomized treatment period with aprocitentan (25 mg or 12.5 mg) or placebo, a 32-week single-blind treatment period with aprocitentan (25 mg), and another 12-week double-blind withdrawal treatment period in which patients were rerandomized to aprocitentan (25 mg) or placebo [59, 60•]. The primary efficacy endpoint was change from baseline to week 4 in SBP measured by unattended automated office BP measurement; secondary endpoints were change in SBP from week 36 to week 40 and 24-h SBP and DBP measured by ambulatory BP monitoring at weeks 4 and 40 [59, 60•].

Results showed statistically significant and clinically meaningful reductions in SBP at week 4 in both aprocitentan dose groups versus placebo, and the primary efficacy endpoint was met [60•]. Least-squares mean (standard error) changes in SBP at 4 weeks were –15.3 (0.9) mm Hg for aprocitentan 12.5 mg, –15.2 (0.9) mm Hg for aprocitentan 25 mg, and –11.5 (0.9) mm Hg for placebo, amounting to respective differences versus placebo of –3.8 (1.3) mm Hg (97.5% CI, − 6.8 to –0.8; *P* = 0.0042) and –3.7 (1.3) mm Hg (–6.7 to –0.8; *P* = 0.0046). Least-squares mean (standard error) changes in office SBP from withdrawal baseline (week 36) to week 40 were –1.5 (0.8) mm Hg for aprocitentan 25 mg and + 4.4 (0.8) mm Hg for placebo (difference –5.8 [1.1]; 95% CI, –7.9 to –3.7; *P* < 0.0001). Placebo-corrected measurements from 24-h ABPM confirmed the significant reductions in SBP at 4 weeks with aprocitentan 12.5 mg (–4.2 mm Hg; 95% CI, –6.2 to –2.1) and 25 mg (–5.9 mm Hg; 95% CI, –7.9 to –3.8); reductions in DBP were comparable to those for SBP. Changes in mean 24-h ABPM measurement from withdrawal baseline (week 36) to week 40 also showed increased SBP (6.5 mm Hg; 95% CI, 4.6 to 8.5) and DBP (6.8 mm Hg; 95% CI, 5.5 to 8.0) with placebo versus aprocitentan. Reductions in BP were maintained over 48 weeks. Aprocitentan was generally well tolerated, with the primary adverse effect being edema. During the double-blind period, edema was reported by 9.1% and 18.4% of patients in the aprocitentan 12.5 mg and 25 mg groups, respectively, compared with 2.9% of patients in the placebo group; during the double-blind withdrawal period, edema was reported by 2.6% of patients receiving aprocitentan 25 mg and 1.3% of patients receiving placebo [60•]. A long-term favorable safety profile is especially important for chronic antihypertensive treatment to be used in patients who often have several comorbidities and are treated with multiple pharmacologic therapies.

## Summary

In the United States, there are clearly documented secular declines in BP control that can be attributed to multiple factors including therapeutic inertia by failing to implement drug therapy in patients consistently exceeding evidence-based BP thresholds; when drug therapy is initiated it is typically of inadequate intensity, as approximately 40% of drug-treated hypertensives and drug-treated uncontrolled hypertensives have been prescribed monotherapy [17•]. Also, patient non-adherence to prescribed antihypertensive medications appears to be highly prevalent in patients with aTRH. This implies that some patients with aTRH are actually undertreated due to medication non-adherence despite the prescription of adequate therapy.

While improved use of existing antihypertensive medications is imperative, new treatment strategies aimed at novel therapeutic targets that combine safely and effectively with existing drug therapies are needed. New treatment options for RHT are being investigated in ongoing clinical trials, targeting novel physiological pathways to effectively lower BP with the potential for incremental protection of pressure-sensitive target-organs beyond what is attributable to BP.

The ERA class of medications is especially promising given the role of ET-1, the most potent known endogenous vasoconstrictor, in the pathogenesis of hypertension. The PRECISION trial of aprocitentan was a rigorously designed study in patients with verified RHT that compared two aprocitentan doses over two randomization periods [60•]. A high proportion of study patients had comorbidities, including diabetes, obesity, previous stroke, and renal dysfunction that have been shown to confer pharmacological treatment resistance to antihypertensive drug therapy. Aprocitentan was well tolerated and superior to placebo in lowering BP at week 4, with a sustained effect at week 40 [60•]. Ideal emerging therapies will safely lower BP to a clinically significant degree, protect pressure-sensitive target-organs in patients with selected co-morbid conditions (eg, CKD, heart failure), have minimal side effects and will avoid deleterious drug interactions with commonly used evidence-based antihypertensive drug therapies.
